# An evaluation of the infection control potential of a UV clinical podiatry unit

**DOI:** 10.1186/1757-1146-7-17

**Published:** 2014-02-28

**Authors:** Paul N Humphreys, Chris S Davies, Simon Rout

**Affiliations:** 1 Hygiene and Disinfection Centre, School of Applied Sciences, University of Huddersfield, Queensgate, Huddersfield, UK; 2 Division of Podiatry and Clinical Sciences, School of Human and Health Sciences, University of Huddersfield, Queensgate, Huddersfield, UK

**Keywords:** Infection control, UV, Bacteria, Fungi, Dermatophytes, Contamination

## Abstract

**Background:**

Infection control is a key issue in podiatry as it is in all forms of clinical practice. Airborne contamination may be particularly important in podiatry due to the generation of particulates during treatment. Consequently, technologies that prevent contamination in podiatry settings may have a useful role. The aims of this investigation were twofold, firstly to determine the ability of a UV cabinet to protect instruments from airborne contamination and secondly to determine its ability to remove microbes from contaminated surfaces and instruments.

**Method:**

A UV instrument cabinet was installed in a University podiatry suite. Impact samplers and standard microbiological techniques were used to determine the nature and extent of microbial airborne contamination. Sterile filters were used to determine the ability of the UV cabinet to protect exposed surfaces. Artificially contaminated instruments were used to determine the ability of the cabinet to remove microbial contamination.

**Results:**

Airborne bacterial contamination was dominated by Gram positive cocci including *Staphylococcus aureus*. Airborne fungal levels were much lower than those observed for bacteria. The UV cabinet significantly reduced (p < 0.05) the observed levels of airborne contamination. When challenged with contaminated instruments the cabinet was able to reduce microbial levels by between 60% to 100% with more complex instruments e.g. clippers, remaining contaminated.

**Conclusions:**

Bacterial airborne contamination is a potential infection risk in podiatry settings due to the presence of *S. aureus*. The use of a UV instrument cabinet can reduce the risk of contamination by airborne microbes. The UV cabinet tested was unable to decontaminate instruments and as such could pose an infection risk if misused.

## Introduction

Infection control is a key issue in podiatry as it is in all forms of clinical practice [1,2]. Infection control studies focussed on podiatry practices have demonstrated the importance of effective disinfection [3-5] and laundry processes [6,7] in the reduction of environmental microbial contamination. This is important since environmental contamination is recognised as a source of healthcare associated infections (HAI) [8,9] and a number of bacterial pathogens have significant survival times on inanimate surfaces [10]. Airborne transmission is also potentially important [11], with pathogens such as Methicillin Resistant *Staphylococcus aureus* (MRSA) able to survive and be transported on skin scales [11]. Airborne contamination is a particular issue in podiatry due to the generation of particulate materials during processes such as drilling [12,13].

Bacterial infections associated with MRSA and *Clostridium difficile* have received considerable attention in the UK due to their dominant role in HAI [14-16]. In the podiatry setting, fungal contamination must also be considered due to dermatophyte infections, where onychomycosis can increase the risk of cellulitis, ulceration and subsequent infection to the elderly, immunocompromised and diabetic [17-19]. Dermatophyte infections are generally transmitted via direct contact with infected individuals, skin and hair debris or contaminated fomites [20]. Fungi including dermatophytes have been shown to survive on laboratory equipment [21], healthcare surfaces [22], veterinary equipment [23], house dust [24] footwear and soft furnishings [25]. In animal studies the airborne transmission of dermatophyte infections has also been observed [26].

Whilst steam sterilisation is the most common practice for instrument decontamination, it has been suggested that UV irradiation should be considered an adjunct to conventional cleaning and disinfection processes [27,28]. Ultraviolet (UV) irradiation has broad spectrum biocidal activity with applications in air, surface and water disinfection [28-31]. UV disinfection has been specifically evaluated against fungi [30,32,33] and has received considerable attention as a treatment for airborne contamination in Healthcare settings [28,34,35]. However, its general use for space disinfection e.g. in operating theatres, has been curtailed by concerns over health risks [19,36,37].

**Figure 1:**
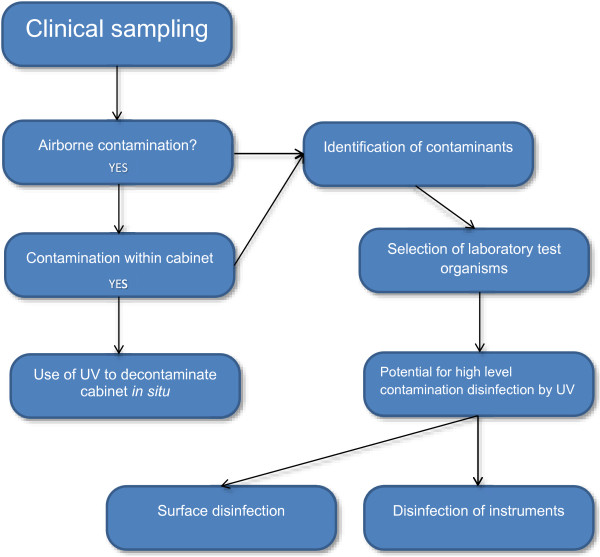
Scheme of work included in the study.

The scheme of work can be seen in Figure 1, the focus of the preliminary section was to determine whether airborne contamination was present during practice within the clinic. If these contaminants were present, the question then arises whether they could be transferred to and survive on surfaces, in particular the cabinet drawer where instruments were stored in the presence and absence of UV. Following identification of the airborne contaminants, representative organisms were selected for assessing the disinfection potential of UV irradiation on surfaces and more complex shapes in the form of instruments from a standard podiatry kit.

## Methods

### Podiatry clinic

**Figure 2:**
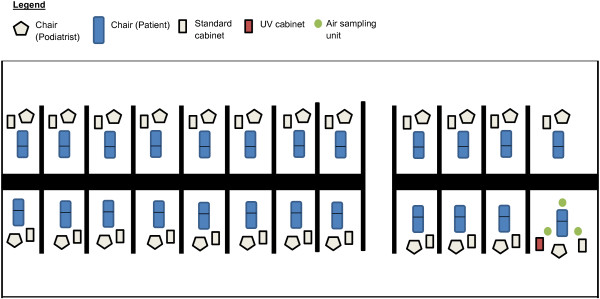
**Schematic of the podiatry clinic sampled in the investigation.** (Not to scale).

All investigations took place in a University podiatry clinic delivering student consultations. The clinic contains a number of podiatry bays each containing a standard podiatry couch and an instrument cabinet (Figure 2). The UV cabinet was placed on the opposite side of the bay from the normal instrument cabinet in an oversized bay designed for the treatment of disabled patients.

### UV podiatry cabinet

**Figure 3:**
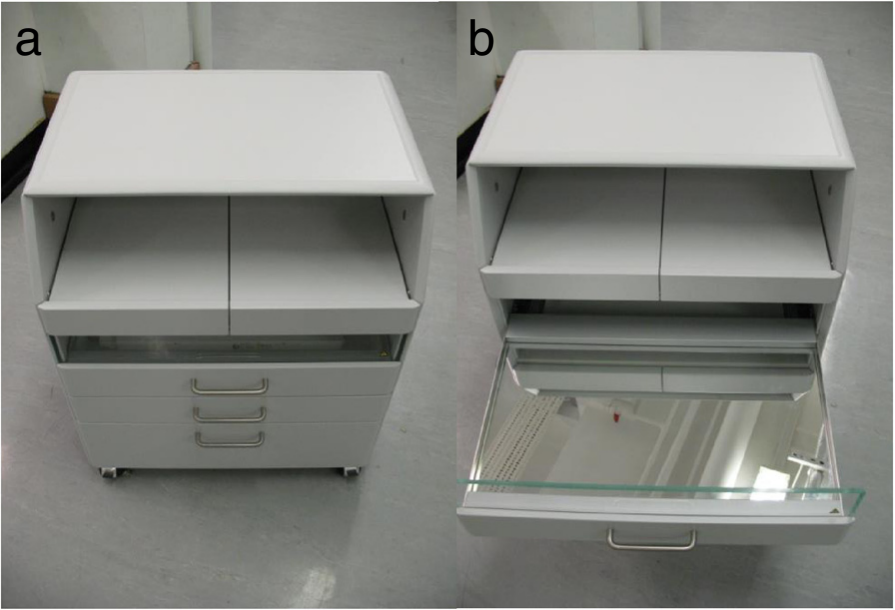
UV podiatry cabinet, UV Drawer closed (a) and open (b).

The podiatry cabinet (Figure 3) is fitted with a retractable drawer with a mirrored base positioned 9 cm below a UV-C emitting tube (Philips TUV 15 watt G15/T8). The drawer has an opaque front to prevent UV leakage and is safety interlocked to prevent the UV tube being activated when the drawer is opened.

### Airborne contamination

To determine levels of microbial air contamination within the Podiatry clinic impact samplers (MicroBio 2, Fred Parrett Ltd, UK) were employed sampling 400 litres of air at a sample rate of 100 l min^-1^. Air samples were taken before, during and after consultations. Bacterial samples were taken on Tryptone Soya Agar (TSA)(LabM Ltd, UK) and were incubated at 37°C for 24 hours; Sabouraud Dextrose agar with Chloramphenicol (SABC) was used for general fungal isolation with Dermasel agar (Oxoid LTD, UK) used to isolate dermatophytes. Fungal plates were incubated at 30°C for up to 6 weeks. In all cases colonies were sub-cultured for analysis.

In addition to air samples, sterile cellulose acetate filters (0.45 μm pore size, Whatman) were employed to determine the ability of the UV cabinet to prevent airborne contamination during routine podiatry consultations. Triplicate filters were placed in sterile Petri dishes located in both the UV cabinet and the standard instrument cabinet and exposed during routine podiatry consultations. After exposure the filters were placed on either TSA, SABC or Dermasel plates and incubated as specified for the air samples.

### Disinfection potential of the UV cabinet

To determine the disinfection potential of the UV cabinet triplicate pre contaminated cellulose acetate filters were exposed to UV radiation from 1 to 30 minutes on two separate days. The filters were contaminated with either *Staphylococcus epidermidis* (NCIMB 12721), *Aspergillus brasiliensis* (ATCC 16404) (previously known as *Aspergillus niger*), *Trichophyton tonsurans* (NCPF 117) or *Trichophyton rubrum* (NCPF 5061). *S. epidermidis* was employed as a surrogate for pathogenic *Staphylococci* such as MRSA. The number of surviving organisms was determined as previously stated with the impact of the UV exposure calculated by comparison with sets of control filters.

*S. epidermidis* contaminated filters were prepared by filtering 0.1 ml of a 10^-3^ dilution of a suspension prepared as specified for *Staphylococcus aureus* in European bactericidal testing standards [38] giving a viable count of between 1.5×10^4^ and 5×10^4^ cfu filter ^-1^. *Trichophyton sp* contaminated filters were prepared by filtering 0.1 ml of a test suspension containing 1.0×10^6^ -2.0×10^6^ spores ml^-1^ recovered from cultures grown on Dermasel plates and recovered as outlined in the European fungicidal testing standard [39].

**Figure 4:**
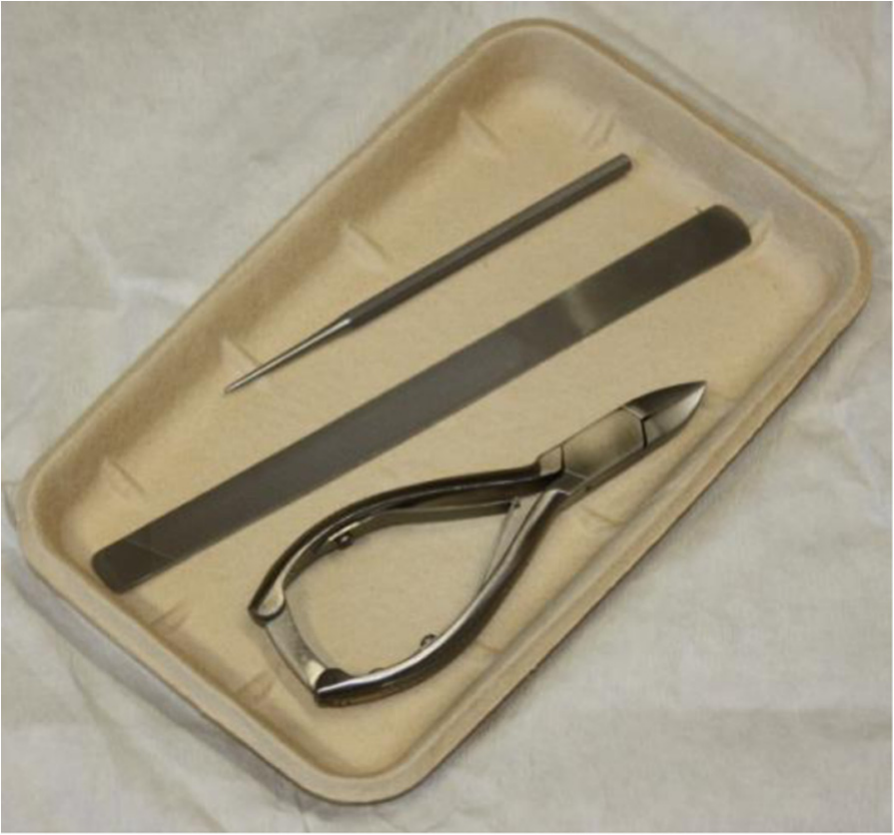
Disposable podiatry instruments.

To investigate the ability of the UV cabinet to decontaminate more complex objects, pre contaminated disposable podiatry instruments were employed. Packs of sterile disposable podiatry instruments (Figure 4) (Vernon Carus Ltd) were unpacked in a Class II microbiological safety cabinet and selected instruments (nail clipper, medium file and the foot dresser) contaminated with either a bacterial or fungal suspension. This was achieved by preparing 1 litre suspensions of inoculum at 1.0-5.0×10^6^ cfu/ml (fungi) or 1.0-5.0×10^8^ cfu/ml (bacteria), which were then transferred to sterile 2 litre containers. The instruments were then completely submerged for 2 minutes, before being allowed to dry for 20 minutes prior to UV exposure in a sterile environment. Triplicate contaminated instruments were UV irradiated for exposure times up to 20 minutes. Following irradiation the number of organisms surviving on the instruments was determined by sonicating in the presence of sterile MRD with 0.05% polysorbate 80 for 5 minutes and then plating on either TSA or Dermasel plates. In addition, a set of control instruments were left in a Class II microbiological safety cabinet for 20 minutes in the absence of UV exposure before being processed in the same manner as treated samples.

### Microbiological analysis

Bacteria isolated during air sampling were characterised using a range of standard microbiological tests [40]. Individual colonies were sub cultured into 96 well plates containing Tryptone Soya Broth (TSB)(Lab M Ltd). Following incubation at 37°C for 24 hours the 96 well plates were replicated onto a range of selective media using 96 well replicators (Sigma Aldrich Ltd) and 150 mm diameter plates. The media employed were chosen to allow the identification of MRSA, *Staphylococcus aureus*, other *Staphylocuccus* spp, *Micrococcus* spp and Gram negative bacilli. The media employed were TSA (aerobic and anaerobic incubation) Mannitol Salt Agar, Baird Parker Agar, Oxacillin Resistant Staphylococci Isolation Medium with methicillin supplement and MacConkeys Agar (all, Lab M Ltd). Presumptive MRSA and *S. aureus* isolates were confirmed via a coagulase latex agglutination test able to detect isolates possessing protein A and/or capsular 5 or 8 antigens. To further aid identification all isolates were Gram stained and tested for catalase and oxidase (both Prolab Diagnostics Ltd).

Fungal isolates recovered on SABC plates were sub cultured onto Malt Extract Agar and identified via microscopic examination. Isolates recovered on Dermasel plates were processed via a dermatophyte PCR kit (SSI, Denmark). PCR was carried out with primers encoding chitin synthase 1 (pan-dermatophytes) and Internal Transcribed Spacer 2 (ITS2) for the detection of *Trichophyton rubrum*. PCR product was run on a 2% agarose gel before being visualised under UV by ethidium bromide staining.

### Statistical analysis

All statistical analysis was carried out using IBM SPSS V20.0.0 for Windows. Data were compared via Analysis of Variance (Anova) and a Tukey HSD post hoc test.

## Results

### Airborne contamination

**Figure 5:**
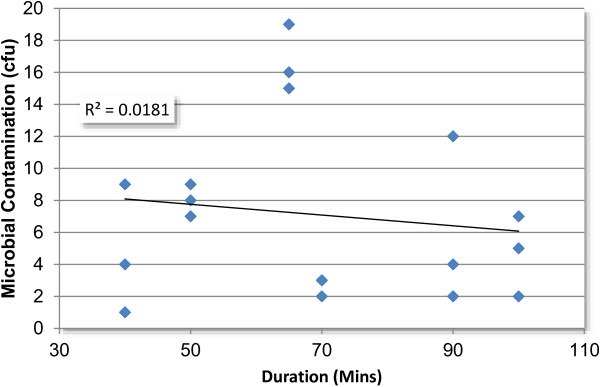
Bacterial air contamination and duration of session.

**Figure 6:**
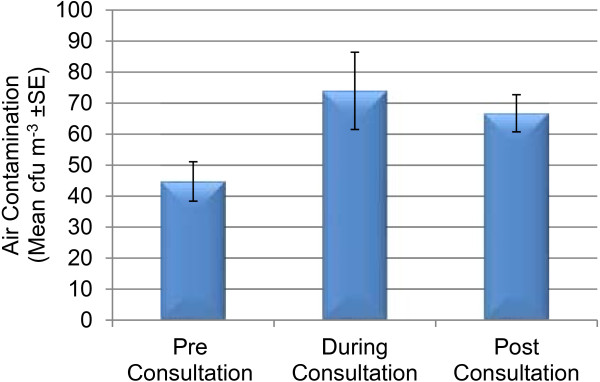
Pre, during and post bacterial air contamination levels.

Over the course of the investigation six student podiatry consultations were sampled for air contamination, these consultations ranged from 40 to 100 minutes with an average of 69 minutes (SE ± 5 minutes). Over the consultation times sampled there was no correlation between the duration of consultation and the level of airborne bacterial contamination detected on the filters exposed on the standard instrument cabinets (Figure 5). Background air sampling indicated that the overall mean level of bacterial air contamination was 58.0 cfu m^-3^ (n = 66, SE ± 4.5). In cases where pre, during and post values were available (n = 18) (Figure 6) no statistical difference was evident between means (p > 0.05). Comparison between the level of contamination detected on the filters exposed in the UV cabinet (n = 18, $\bar{x}$ = 0.11, SE ± 0.08) and those exposed in the instrument cabinet (n = 18, $\bar{x}$ =7.1, SE ± 1.3) indicated that the level of contamination resulting from exposure in the UV cabinet was significantly lower than that resulting from exposure in the standard instrument cabinet (p < 0.05). The exposed filters indicated an average deposition rate of 6.8 cfu hr^-1^ (SE ± 1.3) (3945 cfu m^-2^ hr^-1^, SE ± 730). No MRSA or coagulase positive *Staphylococcus sp* were recovered from the exposed filters.

**Figure 7:**
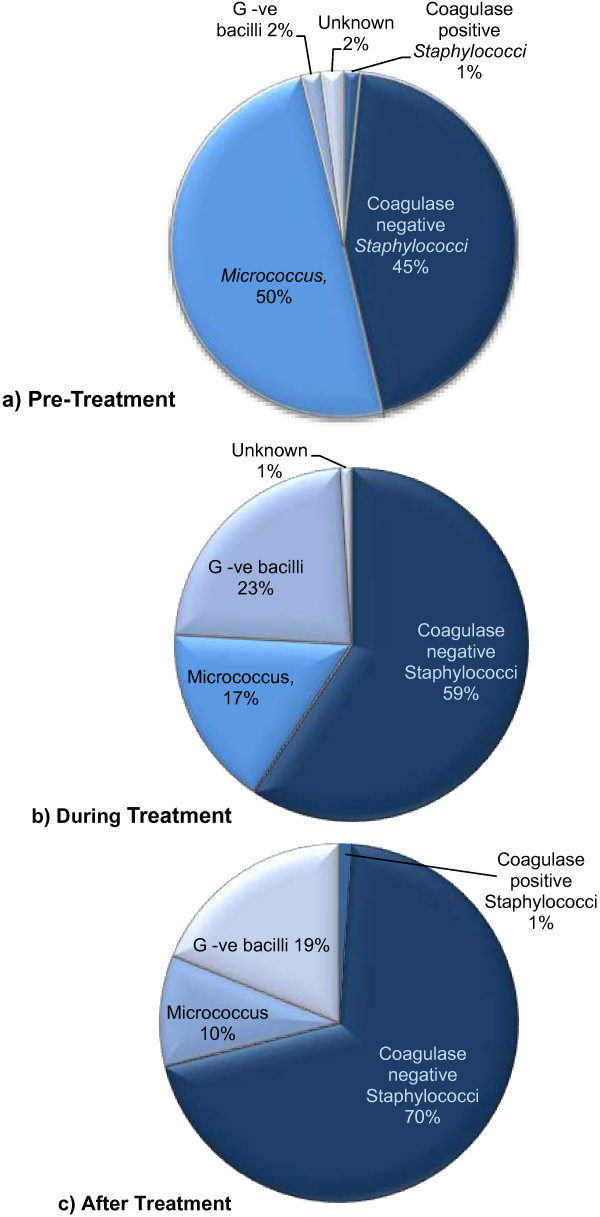
Characterisation of bacterial air contamination a) pre-treatment, b) during treatment and c) after treatment.

Characterisation of bacterial air contamination (Figure 7) indicated that during and post treatment the most common bacteria detected were coagulase negative *Staphylococcus sp*, representing greater than 50% and 70% of isolates respectively. Pre-treatment, *Micrococcus sp* were the most abundant at ≈50%, followed by coagulase negative *Staphylococcus sp* at 45%. The numbers of coagulase positive *Staphylococcus sp* (presumptive *S. aureus*) represented less than 2% across all samples investigated. Gram negative bacilli were present across all three sampling periods, representing 1.8% of colonies pre-treatment, increasing to 23.1% and 18.9% of the totals recovered during and post-treatment. In all cases only 2% of isolates remained unclassified.

**Figure 8:**
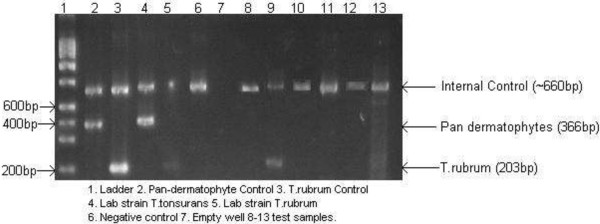
Pan-Dermatophyte PCR test.

In contrast to bacteria, the number of fungi recovered during air sampling was much lower across both media types (SABC/Dermasel). Sampling for general fungi on SABC plates obtained the most fungal colonies, with an overall average of 2.2 cfu m^-3^ (n = 36, SE ± 0.8). Microscopic analysis of fungi recovered from SABC plates indicated that isolates were dominated by *Penicillium* and *Aspergillus* species. Fungi recovered on Dermasel plates indicated an overall mean level of air contamination of <1.0 cfu m^-3^ (n = 36, $\bar{x}$= 0.9, SE ± 0.3). A total of six Dermasel isolates were subjected to further analysis via the SSI dermatophyte PCR kit. Only one of these was confirmed as a dermatophyte (*T. rubrum)*, the remaining isolated samples tested negative to pan-dermatophyte and *T. rubrum* primers (Figure 8). No fungi were recovered from the filters exposed in either the UV or conventional instrument cabinet.

### Disinfection potential

**Figure 9:**
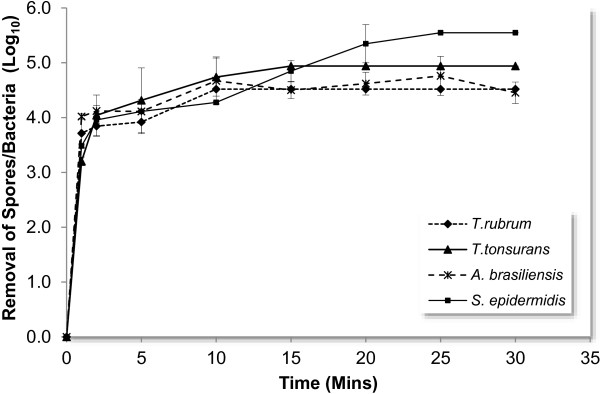
Impact of UV exposure on contaminated filters.

When inoculated cellulose nitrate filters were exposed to UV irradiation, complete removal of all four species was seen in 20 minutes (Figure 9). At the lowest exposure time of 1 minute, a >99.9% removal of inoculum was seen in all species. The UV resistance of *A. brasiliensis* was not appreciably greater than the two *Trichophyton* species investigated consequently no further investigations were carried out on *A. brasiliensis*.

**Figure 10:**
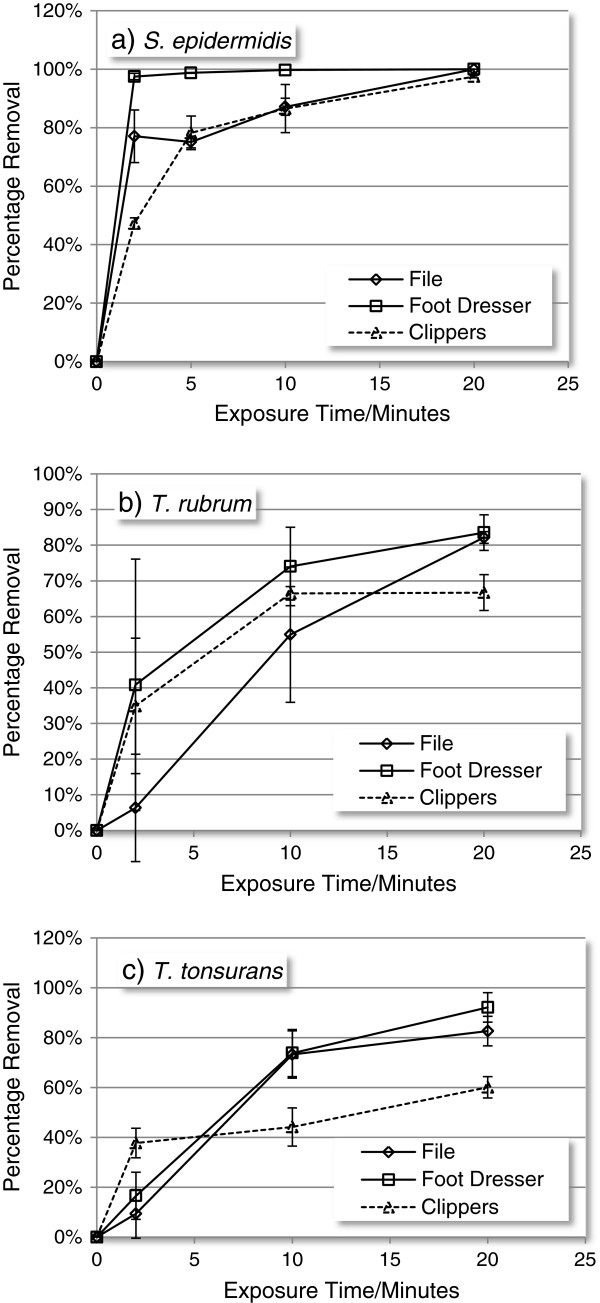
**Impact of UV exposure on instruments contaminated with a) *S. epidermidis*, b) *T. rubrum *and c) *T. tonsurans***.

Given the total removal of contamination from inoculated filters within 20 minutes, this exposure time was then used as the maximum exposure for further investigations with contaminated instruments. In the case of inoculated instruments, contaminant removal increased with UV exposure time across all three instruments (Figure 10a-c). At short exposure times (2 minutes) removal was highly variable, becoming more consistent after 10 minutes exposure. Across all three organisms *T. rubrum* exhibited the greatest variability in survival following UV exposure. In the case of *S. epidermidis* complete removal was achieved for all instruments within 20 minutes (Figure 10a), in the case of dermatophyte spores, removal was least effective in the case of the contaminated clippers (Figure 10b and c). The profiles of spore removal (Figure 10b and c) suggest that the structure of the clippers protected 30 to 40% of spore load from UV exposure.

## Discussion and conclusions

Average levels of airborne bacterial contamination (58.0 cfu m^-3^), were towards the lower end of the range published for a podiatry clinic prior to the application of a filtration system (79.0-117.0 cfu m^-3^), and slightly above those observed following the installation of a filtration unit designed for removal of chemical and microbial contaminants (26.0-57.0 cfu m^-3^) [41]. With a wider scope, the numbers observed within the podiatry clinic fell between those published for empty (16.9 [42], 12.4 [43] cfu m^-3^) and operational (140.1 [42], 93.8 [43], 123.2 [44] cfu m^-3^) operating theatres. The levels were also below average values (116.0 to 165.0 cfu m^-3^) published by an extensive study of air conditioned office buildings in the USA. These levels are within the range published by the DoH for operating theatres, and below the maximum level expected in a working operating theatre [45]. The relatively low average levels of airborne contamination experienced in the podiatry clinic may reflect the limited exposure to patient based activity in each cubicle when compared to busy hospital ward or out-patient clinical environments. Consultations carried out in the clinic studied required limited use of curtains, avoiding redistribution of contaminants by disturbing settled organisms as seen in previous studies, contributing to lower average values [6]. There was no evidence that the levels of air contamination increased during consultations or that the length of consultation had an impact on contamination levels. This disagrees with previous studies [46] which have shown an increase in bacterial contamination with subsequent consultations, this difference is likely to reflect the improved ventilation employed in more modern podiatry clinics.

The dominance of the airborne microbiota by Gram positive bacteria, particularly cocci, is consistent with culture based [47-49] and molecular investigation [50,51] of indoor [47-49] environments reflecting the human origin of many indoor, airborne bacteria [50-52]. The dominance of coagulase negative *Staphylococcus* and *Micrococcus* sp also agrees with observations from microbiological studies of podiatry cubicle curtains [6]. The presence of coagulase positive *Staphylococci,* (presumptive *S. aureus*) albeit at significantly lower concentrations, indicates that airborne contamination does pose an infection risk. The risk posed by *S. aureus* in podiatry settings, particularly to diabetic and immunocompromised patients has been recognised by other authors [6]. The same authors [6] also pointed out that the acquired infection rate associated with podiatry was considered to be “virtually nonexistent” although no supporting evidence was available.

The isolation of *Aspergillus* and *Penicillium* sp is also consistent with previous observations [47,48]. The presence of very low numbers of airborne dermatophytes suggests that the risk of air transmission of fungal infections is low but does exist. It is more likely that the cross infection risk associated with dermatophyte infections is greater for contaminated instruments and fomites; this risk being controlled by the sterilisation procedures in place within the clinic for instrument processing.

Given the levels of airborne contamination it is not surprising that filters exposed without the protection of UV irradiation accumulated significantly more bacterial contamination than UV protected filters. The deposition rates are consistent with those associated with other healthcare environments being approximately 50% greater than floor level deposition data from working operating theatres (1790 cfu m^-2^ h^-1^)^48^. The presence of both pathogenic bacteria and fungi in the ambient air indicates that there is the potential for the contamination of sterile instruments placed in the standard sterile field employed by podiatrists. The data indicates that the use of a UV cabinet to store instruments prior to use, may reduce the chance of airborne contamination of instruments within the cabinet drawer and reduce any associated infection risk through patient contact with instruments. The UV cabinet may also remove any contamination due to aerial deposition between uses. However, UV exposure is unable to decontaminate instruments with significant contamination (>4 Log CFU/ml) due to the complex shapes of the instruments which prevent UV penetration. Equally, other decontamination methods have found issues with removing residual proteins from ultrasound and steam processed instruments [5]. This suggests that the mis-use of the cabinet for the recycling of used instruments between patients may present a risk of infection through organism attachment to debris remaining on the instrument from previous disinfection processes [5]. Given the lack of any substantiated data on podiatry associated infections within out-patient based clinic (where no surgery has taken place), it is likely that use of a UV cabinet could only be justified in the case of highly susceptible individuals. Where a cabinet such as this is available local procedure would have to be in place to ensure it is not misused i.e. not use to recycle instruments.

## Competing interests

The authors declare that they have no competing interests.

## Authors’ contributions

PNH designed the study and managed the microbiological aspects of the research. He also contributed to the statistical analysis, data processing and drafting of the manuscript. CSD assisted in the design of the study and managed the podiatry aspects of the research. He also contributed to the data processing and drafting of the manuscript. SR was responsible for the microbiology and molecular aspects of the study. He also contributed to the data processing and initial drafting of the manuscript. All authors read and approved the final manuscript.
